# Apatinib, a Novel Tyrosine Kinase Inhibitor, Promotes ROS-Dependent Apoptosis and Autophagy via the Nrf2/HO-1 Pathway in Ovarian Cancer Cells

**DOI:** 10.1155/2020/3145182

**Published:** 2020-05-13

**Authors:** Xiaodan Sun, Ji Li, Yizhuo Li, Shouhan Wang, Qingchang Li

**Affiliations:** ^1^Department of Pathology, College of Basic Medical Sciences, China Medical University, Shenyang 110122, China; ^2^Department of 2nd Gynecologic Oncology Surgery, Jilin Cancer Hospital, Changchun 130012, China; ^3^Department of Hepatopancreatobiliary Surgery, Jilin Cancer Hospital, Changchun 130012, China; ^4^Department of Pathology, China Medical University, Shenyang 110122, China

## Abstract

Apatinib, a new-generation oral tyrosine kinase inhibitor targeting the vascular endothelial growth factor receptor 2 (VEGFR2) signaling pathway, shows favorable therapeutic effects in various malignant tumors. However, its effect on ovarian cancer has not yet been characterized. Here, we demonstrated that apatinib inhibited ovarian cancer cell growth and migration in a concentration-dependent manner. Further, we found that apatinib could directly act on tumor cells and promote ROS-dependent apoptosis and autophagy. Mechanistically, we showed that apatinib suppressed glutathione to generate ROS via the downregulation of the nuclear factor erythroid 2-related factor 2 (Nrf2)/heme oxygenase 1 (HO-1) pathway and maintained an antitumor effect at a low level of VEGFR2 in ovarian cancer, suggesting that combination of apatinib with Nrf2 inhibitor may be a promising therapy strategy for patients with ovarian cancer.

## 1. Introduction

Ovarian cancer (OC) ranked eighth in incidence and seventh in mortality rates globally among all cancers in women in 2018 (WHO, http://gco.iarc.fr/today/home); it has become the leading malignancy in gynecological cancers in China, with an estimated 52,100 new cases and 22,500 deaths in 2015 [1]. The standard regimen for advanced OC is platinum-based chemotherapy following debulking surgery. However, approximately 75% of patients with advanced stages will eventually experience recurrence [2], and almost all patients with recurrent disease ultimately develop platinum resistance, resulting in poor prognosis with only 40% of patients surviving for 5 years [3]. As such, improved treatment options for OC are urgently needed.

Angiogenesis is universally considered a cancer hallmark and is responsible for tumor proliferation, progression, and metastasis [4], making its interruption an attractive therapeutic strategy for OC. The vascular endothelial growth factor (VEGF)/VEGF-receptor (VEGFR) signaling pathway is a key regulator of angiogenesis; emerging studies have demonstrated the potent efficacy of anti-VEGF antibodies and VEGFR inhibitors in the treatment of OC [5]. Bevacizumab, a monoclonal antibody against VEGF, is one of the most studied angiogenesis inhibitors; it is approved for the first- and second-line treatments of advanced epithelial OC according to the National Comprehensive Cancer Network Guidelines [6]. Unfortunately, this is an inconvenient and costly treatment that is not attainable for all patients with OC in China.

Apatinib, also known as YN968D1, is a novel oral small-molecule tyrosine kinase inhibitor developed in China. It can block the migration and proliferation of VEGFR-induced endothelial cells and reduce tumor microvascular density via highly selective targeting of VEGFR-2 [7]; it was approved by the Chinese Food and Drug Administration in 2014 as a third-line treatment for patients with advanced gastric or gastroesophageal adenocarcinoma. Increasing evidence indicates that apatinib exerts favorable antitumor effects with tolerable toxicities in other human cancers, including breast cancer [8], non-small-cell lung cancer (NSCLC) [9], colon cancer [10], hepatocellular carcinoma [11], pancreatic cancer [12], anaplastic thyroid cancer [13, 14], and osteosarcoma [15].

To date, there have been limited studies on the therapeutic efficacy of apatinib in patients with OC, and its molecular mechanism in this application has not been characterized. In the present study, we investigated the effect of apatinib in OC and observed that a novel regulatory mechanism could underlie its antitumor effect.

## 2. Materials and Methods

### 2.1. Antibodies and Reagents

The following primary antibodies were purchased from Cell Signaling Technology (Danvers, MA, USA): GAPDH, histone H3, *β*-actin, E-cadherin, N-cadherin, vimentin, matrix metalloproteinase 9 (MMP9), PARP, Bax, Bcl2, P62, light chain 3B (LC3B), VEGFR2, nuclear factor erythroid 2-related factor 2 (Nrf2), heme oxygenase 1 (HO-1), and SOD2. The following secondary antibodies were provided by Proteintech (Wuhan, China): goat anti-rabbit IgG, goat anti-mouse IgG, and FITC-conjugated secondary antibody. Apatinib and TBHQ (an Nrf2-specific activator) were purchased from MCE, China. N-acetyl-L-cysteine (NAC), a reactive oxygen species (ROS) scavenger, was purchased from Selleck, China.

### 2.2. Cell Culture and Treatments

A2780, SKOV-3, and CAOV-3 human OC cell lines were purchased from the China Center for Type Culture Collection (Wuhan, China). A2780 and CAOV-3 cells were cultured in DMEM supplemented with 10% fetal bovine serum (FBS). SKOV-3 cells were cultured in RPMI-1640 medium supplemented with 10% FBS. The cells were grown at 37°C in a humidified atmosphere with 5% CO_2_.

Transient transfection was carried out using Lipofectamine 3000 reagent (Invitrogen, Carlsbad, CA) according to the manufacturer's instructions. A VEGFR2 expression plasmid and the corresponding empty plasmid (OriGene, Rockville, MD, USA) were used for VEGFR2 overexpression and as a negative control, respectively. Cells were transfected with VEGFR2-siRNA and negative control siRNA (GenePharma, Shanghai, China) for VEGFR2 knockdown experiments.

### 2.3. Cell Proliferation and Colony Formation Assays

To evaluate cell proliferation, Cell Counting Kit-8 (CCK-8, Beyotime, Shanghai, China) assay was performed. After seeding in 96-well plates at a density of 5000 cells/well with 100 *μ*l culture medium, the cells were treated with different concentrations of apatinib (0, 1, 5, 10, 20, and 40 *μ*M) for an indicated time (24, 48, 72, and 96 h), changing the apatinib-containing medium every 48 h. At the end of the experiments, 10 *μ*l of CCK-8 reagent was mixed into each well, and the cells were incubated at 37°C for 2 h. The absorbance (OD) of each well was measured at 450 nm using a microplate reader. The percentage of cell viability was calculated as (experimental group OD − blank well OD)/(control group OD − blank well OD) × 100%. GraphPad Prism 7.0 software was used to calculate values indicating 50% inhibition of surviving fraction (IC50).

For the colony formation assays, 500 cells per well were plated in 6-well plates. After coincubation with 0, 1, 5, and 10 *μ*M apatinib for 14 days, the cells were washed three times with PBS, fixed with 4% paraformaldehyde, and stained with Giemsa solution. The number of colonies containing more than 50 cells was counted by using a microscope. Colony formation efficiency was calculated as (colony numbers/500) × 100%.

### 2.4. Transwell and Wound Healing Assay

Twenty-four-well transwell chambers (8 *μ*m pore size, 6.5 mm diameter; Millipore, USA) coated with Matrigel (BD Biosciences, San Jose, CA, USA) were used to perform a cell migration assay. First, 500 *μ*l of medium containing 10% FBS was added to the bottom of the chamber. Next, 100 *μ*l of the OC cell suspensions at a density of 10 × 10^4^ cells/ml in a serum-free medium, treated with different concentrations of apatinib (0, 10, and 20 *μ*M), was seeded into the upper chambers. After 24 h, the cells that adhered to the upper surfaces of the transwell membranes were removed using cotton swabs, and those on the lower surfaces were fixed with 4% paraformaldehyde and stained with a 0.1% crystal violet dye. The migrated cells were photographed and counted in five random fields using an inverted microscope.

To assess wound healing, cells were plated in six-well plates and the confluent monolayer cell plate was wounded using the tip of a 250 *μ*l pipette. PBS was used to remove floating cells, and the cells were cultured in a serum-free medium in the presence or absence of apatinib for 24 h. Images of the same position of the wounded monolayer were obtained by using a microscope. ImageJ software was used to quantitatively measure wound distance.

### 2.5. Analysis of Apoptosis

Annexin V-FITC/PI Apoptosis Detection Kit (BD, Biosciences, China) was used to detect apoptosis. After treatment with the indicated concentrations of apatinib (0, 10, and 20 *μ*M), the harvested cells were resuspended in Annexin V-binding buffer, then stained with FITC-conjugated Annexin V and PI according to the manufacturer's protocol. The degree of apoptosis was analyzed using a flow cytometer (LSRFortessa, BD Biosciences).

### 2.6. ROS Detection and Measurement of Intracellular Glutathione (GSH)

ROS induced by apatinib was determined using a ROS Assay Kit (Beyotime, Shanghai, China) according to the manufacturer's protocol as previously described [16]. Briefly, after exposure to apatinib (0, 10, and 20 *μ*M) for 24 h, the cells were incubated with 10 *μ*M 2′-7′ dichlorofluorescin diacetate (DCFH-DA) in the dark for 20 min at 37°C in a humidified atmosphere at 5% CO_2_. Next, the cells were washed three times with cold PBS to remove excess fluorescent probe. The cells were then observed using a fluorescence microscope or resuspended in 300 *μ*l of PBS and assessed for fluorescence intensity using a flow cytometer (LSRFortessa). The data were analyzed using FlowJo X 10.0.7 Software.

A Total Glutathione Assay Kit (Beyotime) was used to measure intracellular GSH levels according to the manufacturer's instructions as previously described [16]. Briefly, after being cocultured with or without apatinib for 24 h and/or pretreated with 200 *μ*M TBHQ for 4 h to activate the Nrf2 pathway, cells were harvested and lysed in the protein removal solution S provided in the kit. After incubation for 5 min at 4°C, the samples were centrifuged at 12,000 rpm for 10 min at 4°C. The supernatant was treated with assay solution for 25 min at 25°C, and the absorbance at 412 nm was measured using a microplate reader (SpectraMax i3x, Molecular Devices, Sunnyvale, CA). Relative intracellular GSH levels were calculated by normalization to the values of the control group.

### 2.7. Immunofluorescence Staining

Cells were seeded in 20 mm culture plates and cocultured with 20 *μ*M apatinib for 24 h or pretreated with 5 mM NAC for 2 h to inhibit ROS generation, then washed with PBS, fixed with 4% paraformaldehyde for 15 min, and permeabilized in 0.1% Triton X-100 for 5 min. After blocking with 5% bovine serum albumin for 1 h at room temperature, the cells were incubated with primary antibody against LC3B (dilution 1 : 100) overnight at 4°C. Then, FITC-conjugated secondary antibody (dilution 1 : 200) was incubated with the cells for 1 h in the dark at room temperature, and the cells were stained with 4′,6-diamidino-2-phenylindole (DAPI) for 5 min to visualize the nuclei. Images were captured using a fluorescence microscope.

### 2.8. Transmission Electron Microscopy (TEM)

After 24 h apatinib treatment (20 *μ*M) or 2 h NAC pretreatment (5 mM), the cells were washed lightly with PBS, digested with 0.25% trypsin, and centrifuged at 3000 rpm for 10 min at 4°C. The samples were fixed in 3% glutaraldehyde overnight at 4°C for fixation. Then, ultrathin sections (100 nm) were stained with 5% uranyl acetate and Reynold's lead citrate and detected using a TEM (H-7650, Hitachi, Tokyo, Japan).

### 2.9. Western Blotting and Nuclear and Cytoplasm Isolation

Total proteins were isolated from OC cells with or without apatinib treatment. After washing with ice-cold PBS three times, cells were lysed in a lysis buffer supplemented with a cocktail of proteinase inhibitors. Equal amounts of protein (40 *μ*g) from cell extracts were separated using 10% SDS-PAGE and transferred onto 0.45 *μ*m polyvinylidene fluoride (PVDF) membranes (Millipore, Billerica, MA, USA) as previously described [16]. ImageJ software was used to evaluate the gray value of each band.

A Nuclear and Cytoplasmic Protein Extraction kit (Beyotime) was used to isolate the cytosolic and nuclear cell fractions, following the manufacturer's instructions as previously described [16]. Briefly, the collected cells were suspended in ice-cold hypotonic buffer and incubated on ice for 20 min. The extracts were then centrifuged at 12,000 × g for 5 min, and the supernatants were collected as cytosolic fractions. The pellets were washed with ice-cold PBS and resuspended in the lysis buffer, followed by vortexing at the highest speed. These extracts were centrifuged at 12,000 × g for 10 min, and the supernatants were collected as the nuclear fractions.

### 2.10. Quantitative Real-Time PCR Analysis (qRT-PCR)

TRIzol (Invitrogen) was used to extract total RNA from ovarian cancer cells. Reverse transcription was performed as preciously described [17] using PrimeScript RT Master Mix (Takara, Otsu, Japan). qRT-PCR was performed as preciously described using Applied Biosystems Power SYBR Green on a qTOWER2.0 [17], briefly, 10 seconds at 95°C, then 40 cycles at 95°C for 5 seconds and 65°C for 34 seconds. The mRNA ratio of the target genes to GAPDH was calculated using the 2^−*ΔΔ*Ct^ formula. The specific primer sequences are performed as follows:

GAPDH, Forward 5′-CCACCCATGGCAAATTCC-3′, Reverse 5′-GATGGGATTTCCATTGATGACA-3′; VEGFR2, Forward-5′GGACTCTCTCTGCCTACCTCAC-3′, Reverse 5′-GGCTCTTTCGCTTACTGTTCTG-3′; Nrf2, Forward 5′-TCATGATGGACTTGGAGCTG-3′, Reverse 5′-CATACTCTTTCCGTCGCTGA-3′; HO-1, Forward 5′-CCAGGCAGAGAATGCTGAGT-3′, Reverse 5′-GGCGAAGACTGGGCTCTC-3′; GCLC, Forward 5′-ACATCTACCACGCCGTCAAG-3′, Reverse 5′-ACAGGACCAACCGGACTTTT-3′; and GCLM, Forward 5′-GGGGAACCTGCTGAACTG-3′, Reverse 5′-TCTGGGTTGATTTGGGAACT-3′.

### 2.11. Online Database

A series of online databases were implemented as previously described [17]. Briefly, the GEPIA database (http://gepia.cancer-pku.cn/), the Oncomine database (http://www.oncomine.org/), and the Human Protein Atlas database (https://www.proteinatlas.org/) were used to analyze mRNA or protein expression of VEGFR2 in OC and normal tissues, respectively.

### 2.12. Statistical Analysis

All experiments were repeated at least three times, and all results are presented as the means ± standard deviations. Statistical analysis was performed using GraphPad Prism 7.0 software. Statistical significance was determined based on Student's *t*-test or one-way ANOVA; *P* values < 0.05 were considered statistically significant.

## 3. Results

### 3.1. Apatinib Suppressed the Growth of OC Cells

**Figure 1:**
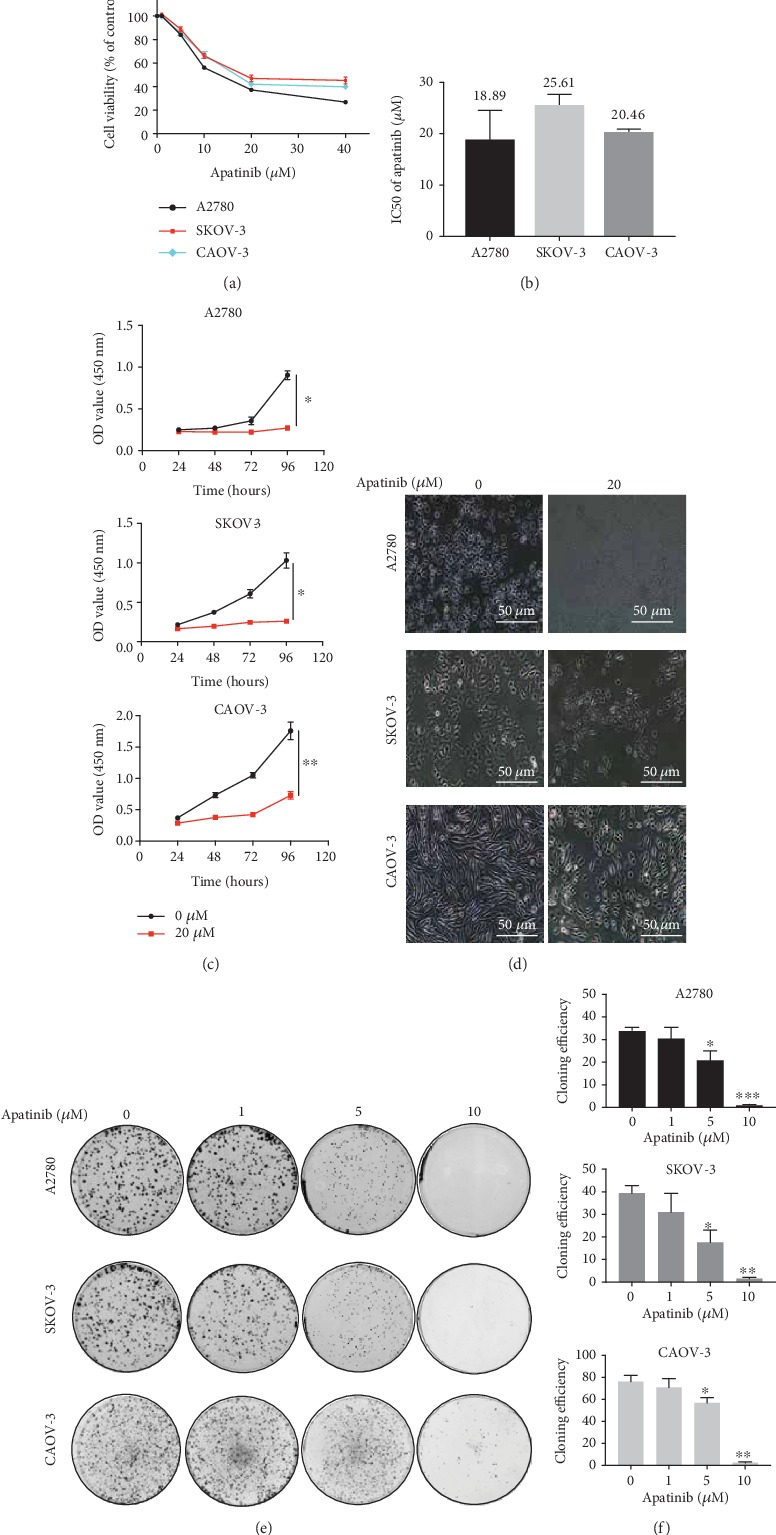
Apatinib suppressed growth of OC cells. (a) Cell viability assays of A2780, SKOV-3, and CAOV-3 cells treated with low-to-high concentrations of apatinib for 48 h. (b) The IC50 values of apatinib for 48 h in three OC cells. (c) The OC cells were treated with 20 *μ*M apatinib for different time intervals (24, 48, 72, and 96 h). The cell viability was detected by CCK-8 and expressed as absorbance value (OD). (d) The effects of 20 *μ*M apatinib on the morphology of OC cells were observed using light microscope (20x). Scale bar = 50 *μ*m. (e, f) Colony formation assay of three OC cells. Colony numbers were counted using the microscope, and the colony formation efficiency was calculated. Data are presented as the mean ± SD of three independent experiments. ^∗^*P* < 0.05, ^∗∗^*P* < 0.01, and ^∗∗∗^*P* < 0.001, compared with the control groups.

First, the cell viability of the A2780, SKOV-3, and CAOV-3 cell lines decreased as the drug concentration increased (Figure 1(a)), with IC50 values of 18.89 ± 5.6, 25.61 ± 2.1, and 20.46 ± 0.5 *μ*M, respectively (Figure 1(b)). These results suggested that apatinib reduced OC cell growth in a concentration-dependent manner. Following this, 50% of the IC50 dose and the IC50 dose, i.e., approximately 10 *μ*M and 20 *μ*M doses of apatinib, were used for the subsequent experiments. Next, we found that the growth of OC cells was suppressed by apatinib in a time-dependent manner as well (Figure 1(c)). In addition, we observed cell morphology changes induced by apatinib. In the control group, A2780 was round, SKOV-3 was epithelial-like, and CAOV-3 was spindle-shaped, in line with previous descriptions of these three OC cell lines [18]. After treatment with apatinib, the number of cells was reduced, and the cells were observed to be smaller and irregular in shape; moreover, they took on indistinct margins, with looser intercellular connections, compared to the cells in the control group (Figure 1(d)). The apatinib-treated cells had a lower colony formation ability than the cells in the control group, especially when 10 *μ*M apatinib was applied (*P* < 0.05; Figures 1(e) and 1(f)). Collectively, these results suggest that apatinib suppressed the proliferation of OC cells in both a concentration- and time-dependent manner.

### 3.2. Apatinib Inhibited OC Cell Migration

**Figure 2:**
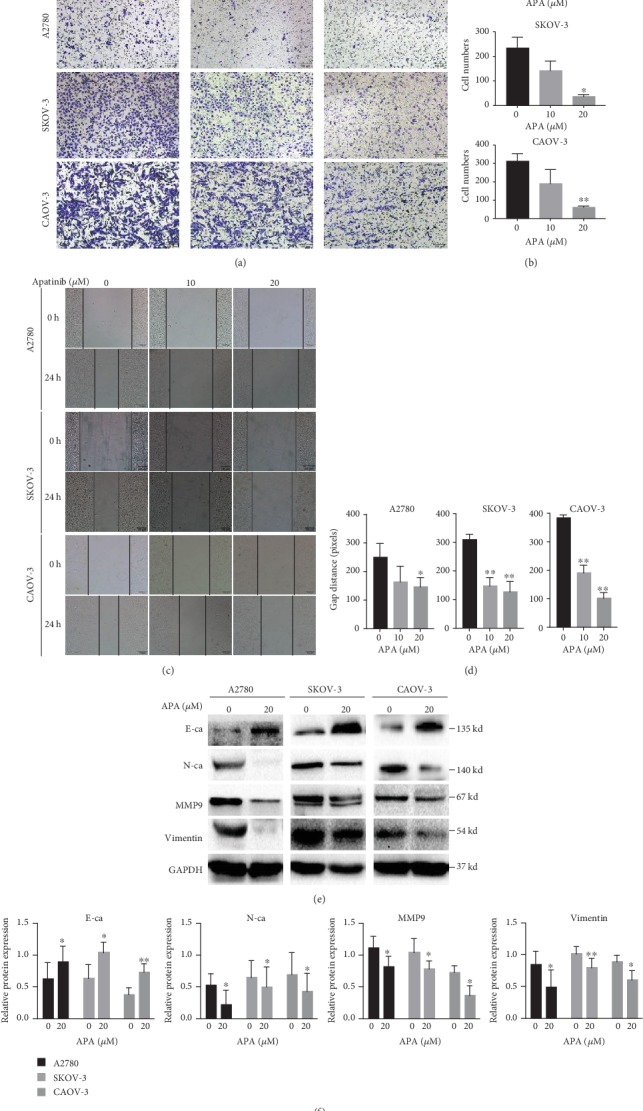
Apatinib inhibited OC cell migration. (a, b) The migration of A2780, SKOV-3, and CAOV-3 cells after treatment with apatinib (0, 10, and 20 *μ*M) for 24 h was assessed using the transwell assay. The invaded cells on the bottom surface of the filters were stained, and the cell numbers were counted by ImageJ software. (c, d) The movement ability of three OC cells after treatment with apatinib (0, 10, and 20 *μ*M) for 24 h was detected using wound healing assays. The gap distance that was calculated by ImageJ software was used to measure the movement ability. (e) After treatment with 20 *μ*M apatinib for 24 h, protein levels of the EMT markers E-cadherin, N-cadherin, vimentin, and metastasis-associated protein, MMP9, in three OC cells were determined by western blotting. (f) The relative western blot gray values are shown in the histogram. Data are presented as the mean ± SD of three independent experiments. APA: apatinib.^∗^*P* < 0.05, ^∗∗^*P* < 0.01, compared with the control groups.

Apatinib is known to specifically inhibit VEGFR2 to suppress tumor angiogenesis, which plays an important role in tumor metastasis. Therefore, we explored the role of apatinib in OC migration using the transwell assay. Cell migration was significantly delayed under apatinib treatment in a concentration-dependent manner, especially at 20 *μ*M (Figures 2(a) and 2(b)). Consistently, the wound healing abilities of OC cells were also significantly decreased in a concentration-dependent manner (Figures 2(c) and 2(d)). Furthermore, we performed western blotting to explore whether apatinib suppresses the levels of epithelial-mesenchymal transition- (EMT-) associated markers in OC cells since EMT is closely related to tumor metastasis. Under apatinib treatment, the level of the epithelial marker E-cadherin increased, whereas the levels of the mesenchymal markers vimentin and N-cadherin decreased. The level of another metastasis-associated protein, MMP9, also decreased under apatinib treatment (Figures 2(e) and 2(f)). Thus, apatinib inhibited OC cell migration mainly via suppressing EMT.

### 3.3. Apatinib Induced Apoptosis and Autophagy in OC Cells

**Figure 3:**
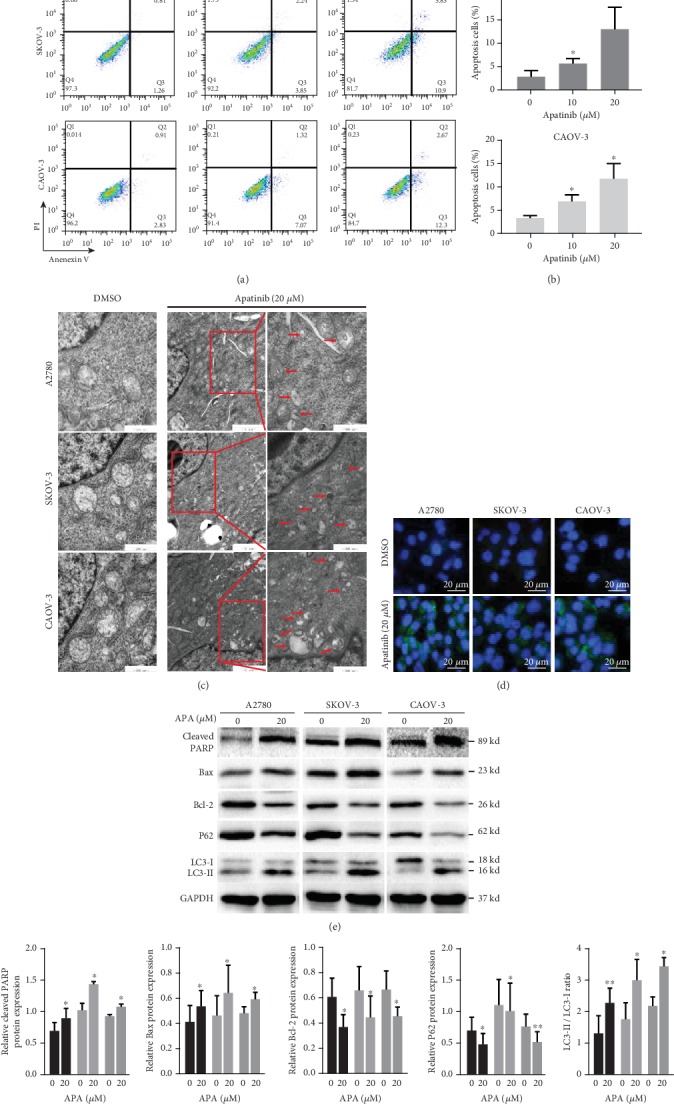
Apatinib induced apoptosis and autophagy in OC cells. (a) After treatment of A2780, SKOV-3, and CAOV-3 cells with apatinib (0, 10, and 20 *μ*M) for 24 h, these cells were stained with Annexin V-FITC/PI and analyzed by a flow cytometer. (b) The quantitative analysis of the apoptotic cell percentages is shown. (c) The representative images of TEM: more autophagic vacuoles (red arrows) are shown in the three apatinib-treated (20 *μ*M) OC cell lines for 24 h compared with the DMSO-treated control groups. Scale bar = 1 *μ*m (the middle panel)/500 nm (the left and right panels). (d) The representative images of immunofluorescence: a dot pattern of LC3-II fluorescence is presented in the apatinib-treated (20 *μ*M) OC cells (40x). Scale bar = 20 *μ*m. (e) After treatment with 20 *μ*M apatinib for 24 h, the protein levels of cleaved PARP, Bcl-2, Bax, p62, and LC3B (LC3 II/LC3 I) were determined by western blotting. (f) The relative western blot gray values are shown in the histogram. Data are presented as the mean ± SD of three independent experiments. APA: apatinib.^∗^*P* < 0.05, ^∗∗^*P* < 0.01, compared with the control groups.

We attempted to identify the potential mechanism underlying the antitumor effect of apatinib. In addition to inhibiting VEGFR2 signal transduction, many studies have shown that apatinib can directly act on tumor cells [10, 12, 14, 15, 19]. Accordingly, we evaluated whether apatinib could induce apoptosis in OC cells. After treatment with 10 and 20 *μ*M of apatinib for 24 h, the percentage of apoptotic cells was significantly higher than that in the control group (*P* < 0.05); this effect was observed to be concentration dependent (Figures 3(a) and 3(b)). In addition to apoptosis, we also tested whether apatinib caused autophagy. After exposure to 20 *μ*M apatinib for 24 h, more autophagosomes with a double membrane containing damaged proteins and organelles and more autolysosomes with a single membrane and degraded contents were observed in the treated group than in the control group using TEM (Figure 3(c)). Next, we evaluated the level of LC3-II, a key marker in the initial stages of autophagy, in OC cells by immunofluorescence. Apatinib-treated cells presented a dot pattern of LC3-II fluorescence, indicating a higher number of autophagosomes than in the control group (Figure 3(d)). In addition, we explored changes in the key indicators of apoptosis and autophagy by western blotting. The levels of cleaved PARP and Bax increased after treatment with 20 *μ*M apatinib, whereas the expression of Bcl-2 decreased. An increase in the conversion of LC3-I to LC3-II, a specific process of autophagy, and a decrease in p62, which is degraded during autophagy, were also detected in apatinib-treated OC cells (Figures 3(e) and 3(f)). These findings suggest that apatinib promoted apoptosis and autophagy in OC cells.

### 3.4. The Generation of ROS Is Crucial for Apatinib-Induced Apoptosis and Autophagy

**Figure 4:**
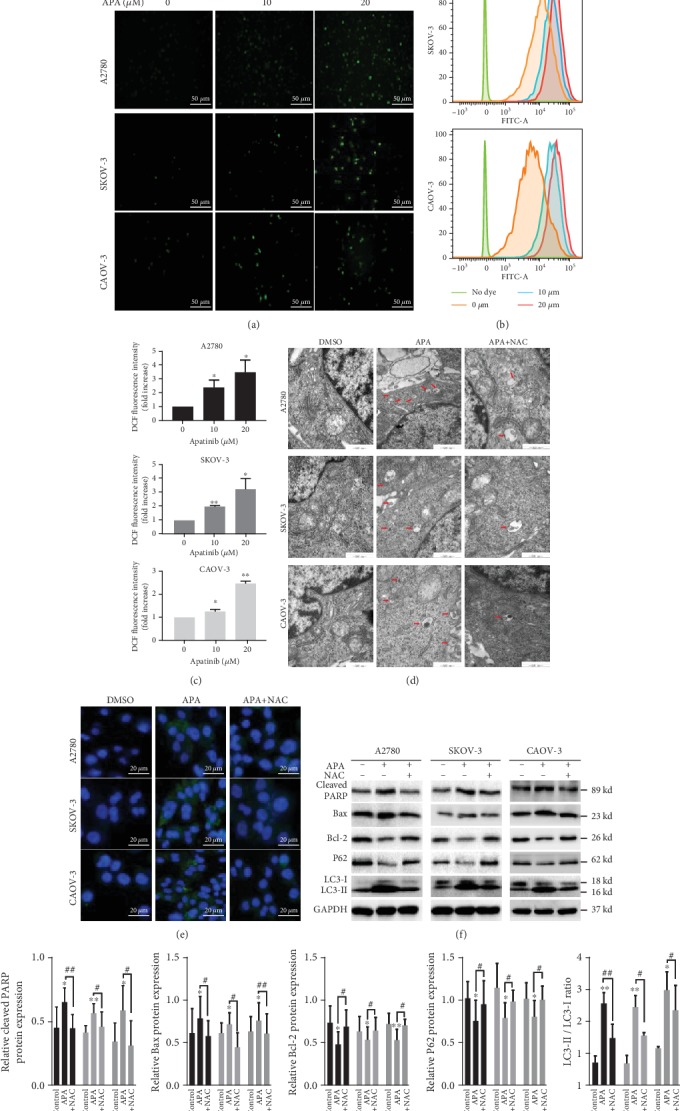
The generation of ROS is crucial for apatinib-induced apoptosis and autophagy. (a) The representative images of fluorescence intensity of DCFH-DA in apatinib-treated (0, 10, and 20 *μ*M) A2780, SKOV-3, and CAOV-3 cells observed by a fluorescence microscope (20x). Scale bar = 50 *μ*m. (b, c) The fluorescence intensity of DCFH-DA was detected by a flow cytometer, and the results were analyzed by FlowJo software. (d, e) The representative images of TEM and fluorescence microscopy after apatinib incubation (20 *μ*M) without or with NAC (5 mM). Scale bar = 20 *μ*m (fluorescence images)/500 nm (TEM images). (f) The expression of apoptosis- and autophagy-related proteins was tested by western blotting after apatinib incubation without or with NAC (5 mM). (g) The relative western blot gray values are shown in the histogram. Data are presented as the mean ± SD of three independent experiments. APA: apatinib.^∗^*P* < 0.05, ^∗∗^*P* < 0.01, compared with the control groups. ^#^*P* < 0.05, ^##^*P* < 0.01.

We further investigated the mechanism by which apatinib promoted apoptosis and autophagy. Since apatinib has been reported to induce ROS in pancreatic cancer and cervical cancer [12, 19] and excessive intracellular levels of ROS may lead to mitochondrial dysfunction to promote apoptosis and autophagy [20, 21], we hypothesized that apatinib induced apoptosis and autophagy by promoting ROS generation. A concentration-dependent increase in the fluorescence intensity of DCFH-DA was observed using a fluorescence microscope in apatinib-treated OC cells compared with the controls (Figure 4(a)); the results were validated by measuring the ROS level using a flow cytometer (Figures 4(b) and 4(c)). As expected, the promotion of apoptosis and autophagy by apatinib was reversed by the administration of NAC (5 mM), a ROS scavenger (Figures 4(d)–4(g)). Collectively, these results indicate that apatinib induced apoptosis and autophagy in a ROS-dependent manner in OC cells.

### 3.5. Apatinib Suppressed GSH to Generate ROS via the Downregulation of Nrf2/HO-1

**Figure 5:**
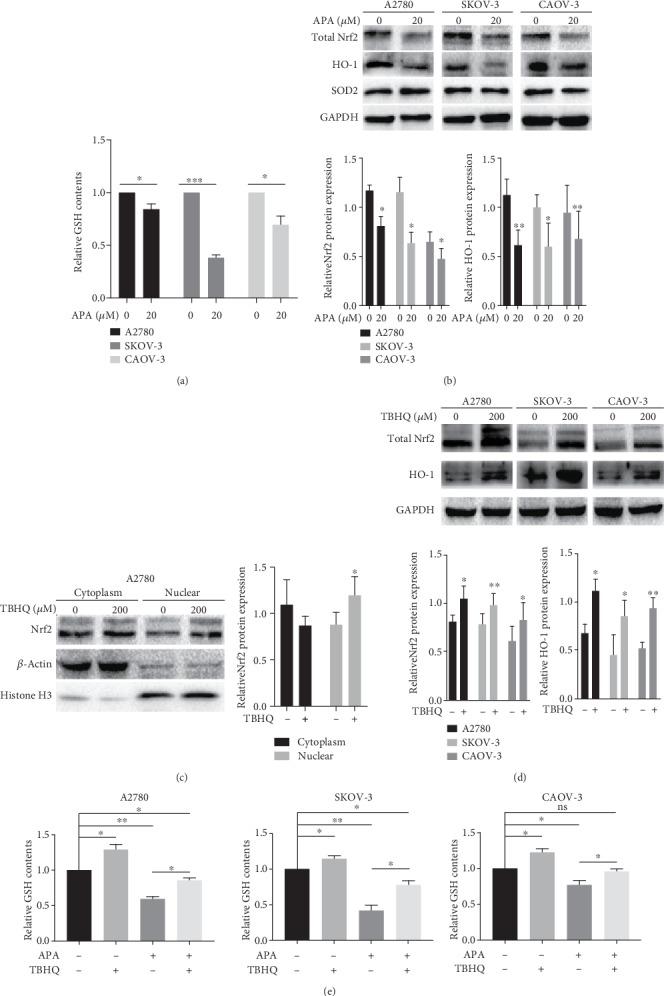
Apatinib suppressed GSH to generate ROS via the downregulation of Nrf2/HO-1. (a) The relative GSH levels in A2780, SKOV-3, and CAOV-3 cells after apatinib treatment (20 *μ*M). (b) The protein expression of Nrf2, HO-1, and SOD2 was tested by western blotting after incubation with apatinib. The relative western blot gray values are shown in the histogram. (c, d) The upregulated protein expression of total Nrf2, nuclear Nrf2, and HO-1 was determined by western blotting and nuclear and cytoplasm isolation after the application of TBHQ (200 *μ*M). The relative western blot gray values are shown in the histogram. (e) The relative GSH levels were detected in the three OC cell lines after incubation in the absence or presence of TBHQ and apatinib. Data are presented as the mean ± SD of three independent experiments. APA: apatinib; ns: not significant. ^∗^*P* < 0.05, ^∗∗^*P* < 0.01, and ^∗∗∗^*P* < 0.001, compared with the control groups.

We subsequently identified the potential molecular mechanisms involved in the generation of ROS by apatinib. First, we measured the levels of GSH, a well-known ROS scavenger [22, 23], in OC cells treated with or without apatinib. As expected, apatinib treatment decreased the level of GSH (Figure 5(a)). Next, we investigated whether apatinib regulates the Nrf2/HO-1 pathway, which is also known to eliminate ROS [24] and is reported to be involved in the regulation of GSH abundance [22]. We found that apatinib decreased the level of Nrf2 and HO-1, whereas SOD2 expression was not significantly changed in apatinib-treated cells as observed by western blotting (Figure 5(b)). These results suggest that apatinib could inhibit the levels of GSH, Nrf2, and HO-1 in OC cells.

To provide further supporting evidence, TBHQ, a specific activator of Nrf2, was utilized. The levels of nuclear and total Nrf2 and HO-1 were significantly upregulated upon the administration of TBHQ, which confirmed the activating effect of TBHQ on Nrf2. (Figures 5(c) and 5(d)). GSH levels were then measured after treating the cells in the absence or presence of TBHQ and apatinib. The results confirmed that the activation of the Nrf2 pathway could upregulate the GSH levels in the cells without apatinib treatment and that the inhibition of GSH by apatinib was reversed by TBHQ treatment (Figure 5(e)). Thus, apatinib could suppress GSH to generate ROS by negatively regulating the Nrf2/HO-1 pathway.

### 3.6. VEGFR2 Regulates Nrf2 Pathway and Apatinib Remains Effective at Low Level of VEGFR2 in OC

**Figure 6:**
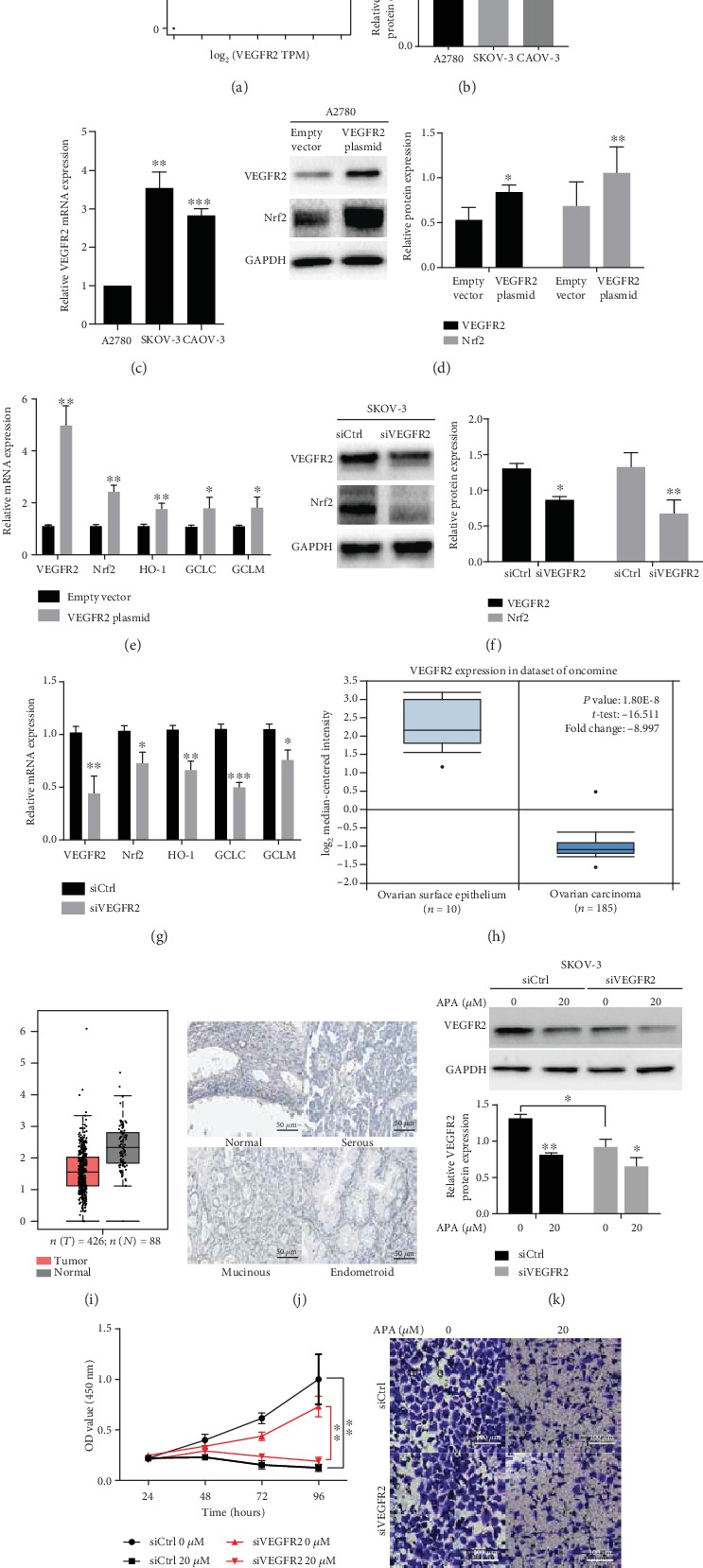
VEGFR2 regulates the Nrf2 pathway, and apatinib remains effective at a low level of VEGFR2 in OC. (a) *Nrf2* had a significant positive relationship with *VEGFR2* based on the GEPIA database. (b, c) The protein and mRNA levels of VEGFR2 were examined in three ovarian cancer cell lines by western blotting and qRT-PCR, respectively. A2780 cells were transfected with VEGFR2 or empty plasmid. (d) The protein levels of VEGFR2 and Nrf2 were tested by western blotting and (e) the increased mRNA levels of VEGFR2, Nrf2, HO-1, GCLC, and GCLM were determined by qRT-PCR. SKOV-3 cells were transfected with VEGFR2 or control siRNA. (f) The protein levels of VEGFR2 and Nrf2 were tested by western blotting and (g) the decreased mRNA levels of VEGFR2, Nrf2, HO-1, GCLC, and GCLM were tested by qRT-PCR. (h) The mRNA levels of VEGFR2 significantly decreased in OC compared with normal ovarian surface epithelium based on the dataset of Oncomine. (i) VEGFR2 mRNA expression was decreased in OC compared with normal tissues based on GEPIA database. (j) Representative immunohistochemical staining images of VEGFR2 in different pathological subtypes of OC and normal tissues based on the HPA database. Scale bar = 50 *μ*m. SKOV-3 cells were transfected with VEGFR2 or control siRNA. (k) The protein levels of VEGFR2 were reduced after incubation with 20 *μ*M apatinib. (l) The cell viability and (m) the migration of transfected SKOV-3 cells were suppressed by apatinib. Scale bar = 100 *μ*m. The relative western blot gray values are shown in the histogram. Data are presented as the *mean* ± *SD* of three independent experiments. APA: apatinib; TPM: transcripts per million.". ^∗^*P* < 0.05, ^∗∗^*P* < 0.01, and ^∗∗∗^*P* < 0.001, compared with the control groups.

As apatinib is a specific VEGFR2 inhibitor, we wondered about the relationship between VEGFR2 and the Nrf2 pathway. Firstly, we found that there was a significant positive correlation between the *Nrf2* and *VEGFR2* mRNA levels based on the GEPIA database (*R* = 0.2, *P* = 4.1*e* − 05; Figure 6(a)). Then, the protein and mRNA levels of VEGFR2 were examined in three ovarian cancer cell lines. A2780 cell line showed the lowest level among these cell lines while SKOV-3 showed the highest level (Figures 6(b) and 6(c)). These two cell lines were used for further experiments. Overexpression of VEGFR2 in the A2780 cell line resulted in upregulated both mRNA and protein levels of Nrf2, indicating that VEGFR2 regulated Nrf2 at the transcription level. In addition, we found that upregulated VEGFR2 could significantly increase the levels of Nrf2 downstream antioxidant genes HO-1, heavy and light subunits of *γ*-glutamyl cysteine synthetase (GCLC and GCLM, which are important rate-limiting enzymes for GSH synthesis) (Figures 6(d) and 6(e)). The opposite results were observed in the SKOV-3 cell line upon downregulation of VEGFR2 by siRNA (Figures 6(f) and 6(g)). These results suggest that VEGFR2 was a positive regulator of Nrf2 pathway.

Next, we investigated VEGFR2 expression in OC tissues based on a series of online databases since bioinformatics analysis has become a hot research focus. The data from the Oncomine database indicated that *VEGFR2* mRNA expression was lower in OC than in normal tissue. In comparison with 10 samples of normal ovarian surface epithelium, the mRNA levels of *VEGFR2* were significantly lower in 185 cases of ovarian carcinoma (*P* = 1.8*e* − 08; Figure 6(h)), and *VEGFR2* was significantly downregulated in different pathological subtypes of OC (serous, mucinous, endometrioid, and clear cell), especially in the serous type (Table 1). The GEPIA database validated the aforementioned results (Figure 6(i)). In addition, the representative immunochemistry images from the HPA database showed that no significantly positive VEGFR2 staining could be detected in any pathological subtype of OC compared with normal tissues (Figure 6(j)). These findings attracted our interest that whether apatinib could function through a low level of VEGFR2 in OC. We confirmed that apatinib could still reduce the level of VEGFR2 in the SKOV-3 cell line when VEGFR2 was downregulated by siRNA (Figure 6(k)). In addition, compared to the control groups, apatinib still exerted inhibitory effect on cell viability and migration although VEGFR2 level was reduced in OC cells (Figures 6(l) and 6(m)). Besides, we previously observed the antitumor effect of apatinib on the A2780 cell line, which expressed a relative low level of VEGFR2 originally. These findings indicate that apatinib remained effective at low level of VEGFR2 in OC.

## 4. Discussion

Apatinib, a small-molecule selective tyrosine kinase inhibitor of VEGFR-2, is considered a new-generation oral antiangiogenesis drug in China; it has been reported to be effective in various solid tumors. In line with previous case reports that apatinib has potential antitumor activity in patients with OC [25–30], we found it to be effective against OC cell proliferation in both a time- and concentration-dependent manner in vitro. Similarly, apatinib has been found to inhibit cell growth in breast cancer [8], NSCLC [9], colon cancer [10], hepatocellular carcinoma [11], pancreatic cancer [12], anaplastic thyroid cancer [13, 14], osteosarcoma [15], and OC [31]. Interestingly, apatinib shows contrasting effects on OC cell proliferation, with no cytotoxic effects on OC cells and no alteration of the cell cycle or apoptosis in vitro. However, it inhibits EMT in OC cells by inhibiting the JAK/STAT3 and PI3K/Akt signaling pathways, resulting in the suppression of tumor volume in vivo by inhibiting tumor angiogenesis [32]. Consistently, we found that apatinib substantially inhibited the migration of OC cells in a concentration-dependent manner by the negative regulation of EMT. This antimetastatic effect of apatinib has also been observed in other cancers, such as breast cancer, colon cancer, pancreatic cancer, and anaplastic thyroid cancer [8, 10, 12, 14]. Thus, apatinib exerts favorable antitumor efficacy and could be a promising therapeutic strategy for patients with OC.

With regard to the antitumor mechanism of apatinib, recent studies have shown that apatinib could act directly on tumor cells by inducing apoptosis and cell cycle arrest [10, 12, 14, 15, 19]. Our results showed that it induced apoptosis in OC cells in a concentration-dependent manner. Likewise, autophagy appears to be involved as more autophagosomes and autolysosomes were present in the treated groups than in the controls. The dot pattern of LC3-II fluorescence, the increased LC3-II/LC-I expression ratio, and a decrease in p62 levels were observed in apatinib-treated OC cells indicating that apatinib can induce autophagy. Interestingly, the contrary roles of apatinib-induced autophagy have been reported. In colon cancer and pancreatic cancer, apatinib promotes tumor cell death and suppresses tumor growth by inducing autophagy [10, 12]. Conversely, apatinib promotes protective autophagy in anaplastic thyroid cancer and osteosarcoma. Moreover, the inhibition of autophagy sensitizes these tumor cells to apatinib-induced apoptosis in vitro and enhances the effect of apatinib-induced growth inhibition in anaplastic thyroid cancer cells in vivo [13, 15]. Therefore, the role of apatinib-induced autophagy may be context specific and should be further explored in OC.

Increasing evidence has shown that ROS plays an important role in tumors [33]; the interplay between ROS, apoptosis, and autophagy has attracted research attention [20, 21, 34, 35]. Treatment with apatinib may increase ROS in pancreatic cancer and cervical cancer [12, 19]. We likewise observed that apatinib promoted ROS generation in a concentration-dependent manner. In addition, apatinib-induced apoptosis and autophagy were ROS-dependent in OC cells, since treatment with a ROS scavenger reversed the promotion effect. Therefore, the promotion of ROS may be the novel cytotoxic effect of apatinib.

Among ROS regulators, GSH is remarkable as it is a ROS scavenger and is involved in multiple processes during tumor development, including cellular proliferation and the development of chemotherapy resistance [22, 23]. The Nrf2/HO-1 pathway, which is well known to eliminate ROS [24], is reported to be involved in the regulation of GSH abundance [22, 36]; moreover, upregulated protein levels of Nrf2 and HO-1 have been found in many tumors, including OC [37, 38]. In our study, the levels of GSH, Nrf2, and HO-1 were significantly inhibited by apatinib and the activation of the Nrf2 pathway upregulated the levels of GSH. The reversal of the apatinib-mediated GSH inhibition by TBHQ indicated that apatinib suppressed GSH to generate ROS by negatively regulating the Nrf2/HO-1 pathway.

In addition, a significant positive correlation was indicated between Nrf2 and VEGFR2 in OC based on the GEPIA database. We confirmed that the levels of Nrf2 and its downstream genes could be significantly regulated by VEGFR2. Among these downstream genes, GCLC and GCLM are two catalytic subunits of glutamate cysteine ligase (GCL), whose level and enzymatic activity constitute rate-limiting steps for GSH synthesis and can also be controlled by Nrf2 [39]. Considering the fact that high GCLC or GCLM levels are found in patients with various cancers [40, 41] and that the reduction of GSH production by the irreversible GCL inhibitor promotes apoptosis and attenuates cell growth in cancer cells [42], we speculate that apatinib may also function as a ROS inducer by suppressing the VEGFR2-Nrf2 path, leading to decreased GCLC and GCLM levels, resulting in the reduction of GSH.

Recent studies have mostly focused on apatinib inhibiting VEGFR2 and its downstream pathway as antiangiogenesis and antitumorigenesis targets [14, 43, 44] owing to the fact that VEGFR2 expression is upregulated in the vasculature of some tumors compared to the normal vascular system [45–47]. For instance, VEGFR2 expression is elevated in osteosarcoma and cervical cancer tissues [15, 19]. Apatinib can inhibit tumor cell growth by blocking the VEGFR2/STAT3/Bcl-2 or Akt/GSK3*β*/angiogenin signaling pathways, suppressing tumor angiogenesis in osteosarcoma and anaplastic thyroid cancer, respectively [14, 15]. Interestingly, in our study, we found that both the mRNA and protein levels of VEGFR2 were lower in OC than in normal tissue based on a series of online databases. Similarly, VEGFR2 was reported to be negatively expressed in breast cancer [48]. However, even though we downregulated the level of VEGFR2 in the SKOV-3 cell line, which showed a relatively high level of VEGFR2, apatinib still exerted effective inhibition on VEGFR2 and antitumor effect on OC cells. Besides, apatinib showed favorable antitumor efficacy on the A2780 cell line, which expressed a relatively low level of VEGFR2 originally. Taken these findings together, we concluded that apatinib could function through a low level of VEGFR2 in OC.

Preclinical research has suggested that apatinib may reverse multidrug resistance by inhibiting some proteins associated with its development [49–51]. In two recent phase 2 clinical trials, patients with platinum-resistant and platinum-refractory OC benefited from treatment with apatinib alone or in combination with oral etoposide [52, 53]. Our previous study indicated that GSH, Nrf2, and HO-1 were upregulated in cisplatin-resistant OC cell lines [16]. Therefore, we speculate that apatinib may increase the sensitivity of OC cells to cisplatin by inhibiting the Nrf2 pathway as well. However, this hypothesis needs to be tested further.

Unfortunately, there is a lack of clarity on the precise mechanism of the relationship between Nrf2 and VEGFR2, although the GEPIA database indicated a significant positive correlation between them. In addition to the direct suppression of Nrf2 as discussed above, another possibility is that Nrf2 could be regulated by apatinib via downstream signaling of VEGFR2, such as Akt [54], Erk, p38, and protein kinase C [55]. However, this hypothesis will also need to be investigated in future experiments.

## 5. Conclusions

**Figure 7:**
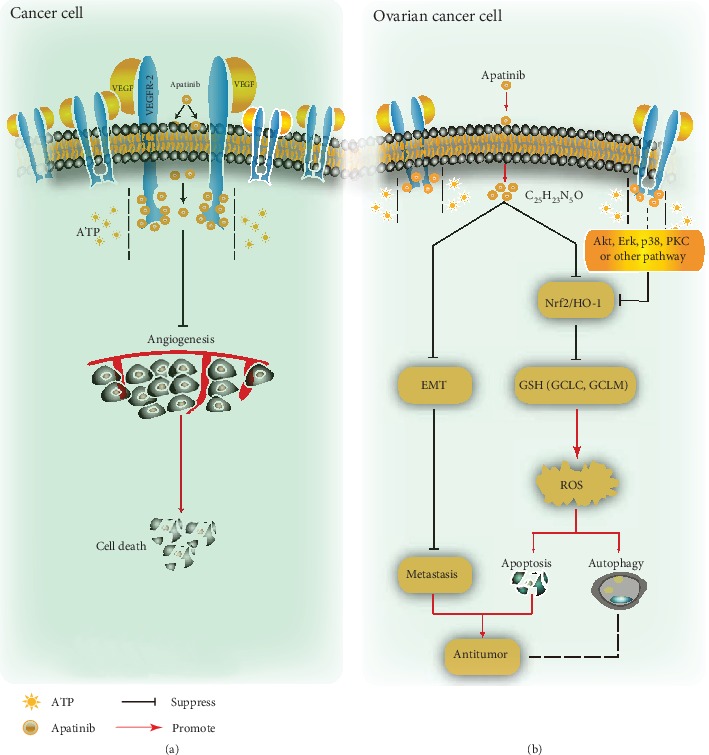
Schematic representation of the antitumor effect of apatinib in cancer cells. (a) After entering the tumor cell, apatinib competes with adenosine triphosphate (ATP) for binding to the ATP site of VEGFR2, inhibiting angiogenesis by blocking downstream signal transduction, resulting in tumor cell death. (b) Apatinib inhibits OC cell migration by suppressing EMT and promotes ROS-dependent apoptosis and autophagy through negatively regulating the VEGFR2/Nrf2/HO-1-GSH pathway in OC cells. PKC: protein kinase C.

In conclusion, we found that apatinib inhibited the proliferation and metastasis of OC cells. Notably, apatinib promoted ROS-dependent apoptosis and autophagy mainly via the inhibition of the Nrf2/HO-1-GSH signaling pathway (Figure 7). This novel regulatory mechanism provides a new perspective for the antitumor effect of apatinib in OC treatment; moreover, the combination of this drug with an Nrf2 inhibitor may be a promising treatment strategy for patients with OC. However, further animal studies or clinical trials need to be performed to confirm this.

## Figures and Tables

**Table 1:** VEGFR2 expression (cancer vs. normal) in other datasets of Oncomine.

Datasets	Subtype	*P* value	*t*-test	Fold change	*N*
Lu ovarian	Serous	**0.007**	-3.921	-1.622	20
	Mucinous	**0.007**	-3.453	-1.592	9
	Endometrioid	**0.009**	-3.514	-1.562	9
	Clear cell	**0.013**	-2.902	-1.480	7

TCGA ovarian	Serous	**0.002**	-3.997	-1.708	586

Yoshihara ovarian	Serous	**8.33E-4**	-3.460	-2.572	22

Adib ovarian	Serous	**0.015**	-3.044	-1.406	6

Hendrix ovarian	Serous	**0.033**	-2.580	-1.137	41
	Mucinous	0.118	-1.313	-1.078	13
	Endometrioid	0.058	-2.075	-1.107	37
	Clear cell	0.157	-1.095	-1.065	8

Notes: the bold font indicates the difference was significant statistically. The negative value of fold change represents that VEGFR2 expression was downregulated. *N*: number of patients.

## Data Availability

The data used to support the findings of this study are available from the corresponding author upon request.

## References

[1] Chen W., Zheng R., Baade P. D. (2016). Cancer statistics in China, 2015. *CA: A Cancer Journal for Clinicians*.

[2] Ozols R. F., Bundy B. N., Greer B. E. (2003). Phase III trial of carboplatin and paclitaxel compared with cisplatin and paclitaxel in patients with optimally resected stage III ovarian cancer: a Gynecologic Oncology Group study. *Journal of Clinical Oncology*.

[3] Jacobs I. J., Menon U., Ryan A. (2016). Ovarian cancer screening and mortality in the UK Collaborative Trial of Ovarian Cancer Screening (UKCTOCS): a randomised controlled trial. *The Lancet*.

[4] Hanahan D., Weinberg R. A. (2011). Hallmarks of cancer: the next generation. *Cell*.

[5] Jackson A. L., Eisenhauer E. L., Herzog T. J. (2015). Emerging therapies: angiogenesis inhibitors for ovarian cancer. *Expert Opinion on Emerging Drugs*.

[6] Matulonis U. A. (2011). Bevacizumab and its use in epithelial ovarian cancer. *Future Oncology*.

[7] Tian S., Quan H., Xie C. (2011). YN968D1 is a novel and selective inhibitor of vascular endothelial growth factor receptor-2 tyrosine kinase with potent activity in vitro and in vivo. *Cancer Science*.

[8] Zhang H., Sun J., Ju W. (2019). Apatinib suppresses breast cancer cells proliferation and invasion via angiomotin inhibition. *American Journal of Translational Research*.

[9] Liu Z. L., Jin B. J., Cheng C. G. (2017). Apatinib resensitizes cisplatin-resistant non-small cell lung carcinoma A549 cell through reversing multidrug resistance and suppressing ERK signaling pathway. *European Review for Medical and Pharmacological Sciences*.

[10] Lu W., Ke H., Qianshan D., Zhen W., Guoan X., Honggang Y. (2017). Apatinib has anti-tumor effects and induces autophagy in colon cancer cells. *Iranian Journal of Basic Medical Sciences*.

[11] Zhang H., Cao Y., Chen Y., Li G., Yu H. (2018). Apatinib promotes apoptosis of the SMMc-7721 hepatocellular carcinoma cell line via the PI3K/Akt pathway. *Oncology Letters*.

[12] He K., Wu L., Ding Q. (2019). Apatinib promotes apoptosis of pancreatic cancer cells through downregulation of hypoxia-inducible factor-1*α* and increased levels of reactive oxygen species. *Oxidative Medicine and Cellular Longevity*.

[13] Feng H., Cheng X., Kuang J. (2018). Apatinib-induced protective autophagy and apoptosis through the AKT-mTOR pathway in anaplastic thyroid cancer. *Cell Death and Disease*.

[14] Jin Z., Cheng X., Feng H. (2017). Apatinib inhibits angiogenesis via suppressing Akt/GSK3*β*/ANG signaling pathway in anaplastic thyroid cancer. *Cellular Physiology and Biochemistry*.

[15] Liu K., Ren T., Huang Y. (2017). Apatinib promotes autophagy and apoptosis through VEGFR2/STAT3/BCL-2 signaling in osteosarcoma. *Cell Death & Disease*.

[16] Sun X., Wang S., Gai J. (2019). SIRT5 promotes cisplatin resistance in ovarian cancer by suppressing DNA damage in a ROS-dependent manner via regulation of the Nrf2/HO-1 pathway. *Frontiers in Oncology*.

[17] Sun X., Wang S., Li Q. (2019). Comprehensive analysis of expression and prognostic value of sirtuins in ovarian cancer. *Frontiers in Genetics*.

[18] Beaufort C. M., Helmijr J. C. A., Piskorz A. M. (2014). Ovarian cancer cell line panel (OCCP): clinical importance of in vitro morphological subtypes. *PLoS One*.

[19] Qiu H., Li J., Liu Q., Tang M., Wang Y. (2018). Apatinib, a novel tyrosine kinase inhibitor, suppresses tumor growth in cervical cancer and synergizes with paclitaxel. *Cell Cycle*.

[20] Li L., Tan J., Miao Y., Lei P., Zhang Q. (2015). ROS and autophagy: interactions and molecular regulatory mechanisms. *Cellular and Molecular Neurobiology*.

[21] Redza-Dutordoir M., Averill-Bates D. A. (2016). Activation of apoptosis signalling pathways by reactive oxygen species. *Biochimica et Biophysica Acta (BBA) - Molecular Cell Research*.

[22] Bansal A., Simon M. C. (2018). Glutathione metabolism in cancer progression and treatment resistance. *Journal of Cell Biology*.

[23] Nunes S. C., Serpa J. (2018). Glutathione in ovarian cancer: a double-edged sword. *International Journal of Molecular Sciences*.

[24] Furfaro A. L., Traverso N., Domenicotti C. (2016). The Nrf2/HO-1 axis in cancer cell growth and chemoresistance. *Oxidative Medicine and Cellular Longevity*.

[25] Cheng Y., Zhang J., Geng H., Qin S., Hua H. (2018). Multiline treatment combining apatinib with toptecan for platinum-resistant recurrent ovarian cancer patients: a report of three cases. *OncoTargets and Therapy*.

[26] Sun H., Xiao M., Liu S., Shi R. (2018). Use of apatinib combined with pemetrexed for advanced ovarian cancer. *Medicine*.

[27] Jin M., Cai J., Wang X., Zhang T., Zhao Y. (2018). Successful maintenance therapy with apatinib inplatinum-resistant advanced ovarian cancer and literature review. *Cancer Biology and Therapy*.

[28] Zhang D., Huang J., Sun Y., Guo Q. (2019). Long-term progression-free survival of apatinib monotherapy for relapsed ovarian cancer: a case report and literature review. *OncoTargets and Therapy*.

[29] Deng L., Wang Y., Lu W., Liu Q., Wu J., Jin J. (2017). Apatinib treatment combined with chemotherapy for advanced epithelial ovarian cancer: a case report. *OncoTargets and Therapy*.

[30] Zhang M., Tian Z., Sun Y. (2017). Successful treatment of ovarian cancer with apatinib combined with chemotherapy. *Medicine*.

[31] Chen L., Cheng X., Tu W. (2019). Apatinib inhibits glycolysis by suppressing the VEGFR2/AKT1/SOX5/GLUT4 signaling pathway in ovarian cancer cells. *Cellular Oncology*.

[32] Ding J., Cheng X. Y., Liu S. (2019). Apatinib exerts anti-tumour effects on ovarian cancer cells. *Gynecologic Oncology*.

[33] Sabharwal S. S., Schumacker P. T. (2014). Mitochondrial ROS in cancer: initiators, amplifiers or an Achilles' heel?. *Nature Reviews Cancer*.

[34] Poillet-perez L., Despouy G., Delage-mourroux R., Boyer-guittaut M. (2015). Interplay between ROS and autophagy in cancer cells, from tumor initiation to cancer therapy. *Redox Biology*.

[35] Scherz-shouval R., Elazar Z. (2011). Regulation of autophagy by ROS : physiology and pathology. *Trends in Biochemical Sciences*.

[36] Kankia I. H., Khalil H. S., Langdon S. P., Moult P. R., Bown J. L., Deeni Y. Y. (2017). NRF2 regulates HER1 signaling pathway to modulate the sensitivity of ovarian cancer cells to lapatinib and erlotinib. *Oxidative Medicine and Cellular Longevity*.

[37] Shim G., Manandhar S., Shin D., Kim T.-H., Kwak M.-K. (2009). Acquisition of doxorubicin resistance in ovarian carcinoma cells accompanies activation of the NRF2 pathway. *Free Radical Biology & Medicine*.

[38] Bao L.-J., Jaramillo M. C., Zhang Z.-B. (2014). Nrf2 induces cisplatin resistance through activation of autophagy in ovarian carcinoma. *International Journal of Clinical and Experimental Pathology*.

[39] Lu S. C. (2013). Glutathione synthesis. *Biochimica et Biophysica Acta (BBA) - General Subjects*.

[40] Fujimori S., Abe Y., Nishi M. (2004). The subunits of glutamate cysteine ligase enhance cisplatin resistance in human non-small cell lung cancer xenografts in vivo. *International Journal of Oncology*.

[41] Lu S. C. (2009). Regulation of glutathione synthesis. *Molecular Aspects of Medicine*.

[42] Andringa K. K., Coleman M. C., Aykin-Burns N. (2006). Inhibition of glutamate cysteine ligase activity sensitizes human breast cancer cells to the toxicity of 2-deoxy-D-glucose. *Cancer Research*.

[43] Fornaro L., Vasile E., Falcone A. (2016). Apatinib in advanced gastric cancer: a doubtful step forward. *Journal of Clinical Oncology*.

[44] Zhao D., Hou H., Zhang X. (2018). Progress in the treatment of solid tumors with apatinib: a systematic review. *OncoTargets and Therapy*.

[45] Plate K. H., Breier G., Millauer B., Ullrich A., Risau W. (1993). Up-regulation of vascular endothelial growth factor and its cognate receptors in a rat glioma model of tumor angiogenesis. *Cancer Research*.

[46] Holmes K., Roberts O. L., Thomas A. M., Cross M. J. (2007). Vascular endothelial growth factor receptor-2: structure, function, intracellular signalling and therapeutic inhibition. *Cellular Signalling*.

[47] Smith N. R., Baker D., James N. H. (2010). Vascular endothelial growth factor receptors VEGFR-2 and VEGFR-3 are localized primarily to the vasculature in human primary solid cancers. *Clinical Cancer Research*.

[48] Holzer T. R., Fulford A. D., Nedderman D. M. (2013). Tumor cell expression of vascular endothelial growth factor receptor 2 is an adverse prognostic factor in patients with squamous cell carcinoma of the lung. *PLoS One*.

[49] Li F., Liao Z., Zhang C. (2018). Apatinib as targeted therapy for sarcoma. *Oncotarget*.

[50] Tong X., Wang F., Liang S. (2012). Apatinib (YN968D1) enhances the efficacy of conventional chemotherapeutical drugs in side population cells and ABCB1-overexpressing leukemia cells. *Biochemical Pharmacology*.

[51] Mi Y.-J., Liang Y.-J., Huang H.-B. (2010). Apatinib (YN968D1) reverses multidrug resistance by inhibiting the efflux function of multiple ATP-binding cassette transporters. *Cancer Research*.

[52] Lan C. Y., Wang Y., Xiong Y. (2018). Apatinib combined with oral etoposide in patients with platinum-resistant or platinum-refractory ovarian cancer (AEROC): a phase 2, single-arm, prospective study. *The Lancet Oncology*.

[53] Miao M., Deng G., Luo S. (2018). A phase II study of apatinib in patients with recurrent epithelial ovarian cancer. *Gynecologic Oncology*.

[54] Ji C., Huang J.-w., Xu Q.-y. (2016). Gremlin inhibits UV-induced skin cell damages via activating VEGFR2-Nrf2 signaling. *Oncotarget*.

[55] Nguyen T., Yang C. S., Pickett C. B. (2004). The pathways and molecular mechanisms regulating Nrf2 activation in response to chemical stress. *Free Radical Biology & Medicine*.

